# Highly accelerated Point-Spread Function mapping based on Finite Rate of Innovation for EPI distortion correction

**DOI:** 10.1186/2197-7364-1-S1-A45

**Published:** 2014-07-29

**Authors:** Rita G Nunes, Joseph V Hajnal

**Affiliations:** Institute of Biophysics and Biomedical Engineering, Faculty of Sciences, University of Lisbon, Lisbon, Portugal; Division of Imaging Sciences and Biomedical Engineering, King’s College London, London, UK; Centre for the Developing Brain, King’s College London, London, UK

**Figure 1:**
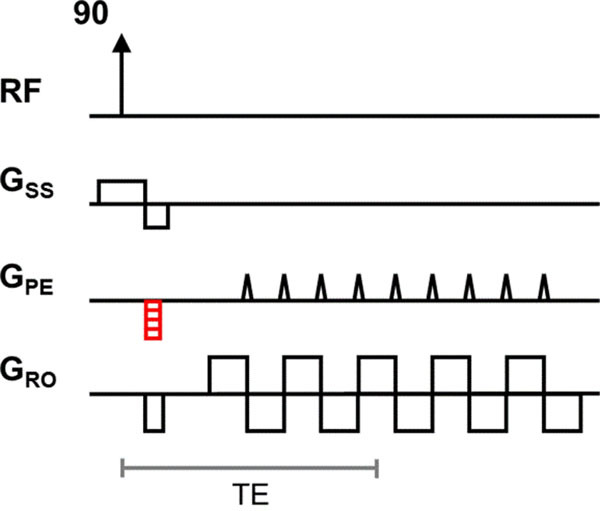
The PSF mapping sequence is repeated while varying the amplitude of the prewinder phase encode gradient (in red).

Hybrid MR-PET scans enable acquisition of both types of images within a session. Despite consistent subject positioning, image alignment can still be challenging. Functional MR images rely on echo-planar imaging (EPI) and present geometric distortions due to static B0 field inhomogeneities. Direct B0 [1] and Point Spread Function (PSF) mapping [2] (Figure 1) have been proposed for distortion correction. The PSF method is more robust [3], but acquisition times are long even with previous acceleration approaches [4–7].

We used the Finite Rate of Innovation (FRI) framework [8, 9] to detect the PSF peak position to sub-pixel precision using as few k_s_ samples as possible.

Images were acquired on a 3.0T Philips Achieva: 2.5×2.5×4.0 mm^3^, matrix 96×95, 95 k_s_ steps with under-sampling retrospectively performed. PSF peak position was estimated using both the fully-sampled (zero-filled by a factor of 1000) and highly-undersampled data.

To determine the PSF peak location, pattern matching was performed. The signal measured at each spatial location was compared to a predicted signal pattern accounting for the k_s_ sampling scheme and the search progressively refined up to the intended precision. EPI images were undistorted as in [10].

**Figure 2:**
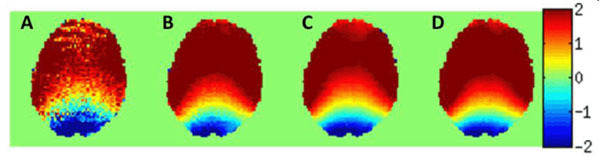
Maps of the PSF shifts (in pixels) estimated with: A0 2; B) 3; C) 4; D) all 95 k_s_

**Figure 3:**
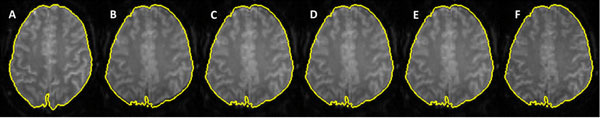
EPI image (with corresponding outer contour in yellow); Undistorted EPI images estimating the displacement field from: B) 2; C) 3; D) 4 and E) all 95 k_s_ samples and F) GE image (corresponding outer contour in yellow propagated to all undistorted EPI images)

Figure 2 shows example PSF peak shift maps (relative to expected undistorted positions). Figure 3A shows the original EPI slice matching Figure 2, and Figures 3B-E show the corrected images using each displacement map. Comparison with the GE image (Figure 3F) confirms accurate geometrical corrections.

Using the proposed approach the position of the PSF peak can be estimated from a very small number of samples. In the future distortion map estimation could easily be incorporated into standard preparation phases. Making distortion correction of EPI images more practical would facilitate combining functional PET and MR information as well as structural connectivity information from diffusion-weighted MR images.
